# Crystal structure of 3-[2-(thio­phen-3-yl)ethyn­yl]-2*H*-chromen-2-one

**DOI:** 10.1107/S2056989015002157

**Published:** 2015-02-07

**Authors:** Ignez Caracelli, Stella H. Maganhi, Hélio A. Stefani, Karina Gueogjian, Edward R. T. Tiekink

**Affiliations:** a Departmento de Física, Universidade Federal de São Carlos, 13565-905 São Carlos, SP, Brazil; b Departamento de Farmácia, Faculdade de Ciências Farmacêuticas, Universidade de São Paulo, 05508-900 São Paulo, SP, Brazil; c Department of Chemistry, University of Malaya, 50603 Kuala Lumpur, Malaysia

**Keywords:** crystal structure, coumarin, asymmetric alkyne, C—H⋯π inter­actions, π–π inter­actions

## Abstract

In the title compound, C_15_H_8_O_2_S, the coumarin moiety is approximately planar (r.m.s. deviation of the 11 non-H atoms = 0.025 Å) and is slightly inclined with respect to the plane of the thio­phen-3-yl ring, forming a dihedral angle of 11.75 (8)°. In the crystal, the three-dimensional architecture features a combination of coumarin–thio­phene C—H⋯π and π–π [inter-centroid distance = 3.6612 (12) Å] inter­actions.

## Related literature   

For the wide range of different biological activities of coumarins, see: Wu *et al.* (2009 ▸); Roussaki *et al.* (2014 ▸). For background to our ongoing inter­est in the synthesis and crystal structures of coumarin derivatives, see: Stefani *et al.* (2012 ▸); Caracelli *et al.* (2015 ▸). For the synthesis of the title compound, see: Gueogjian (2011 ▸).
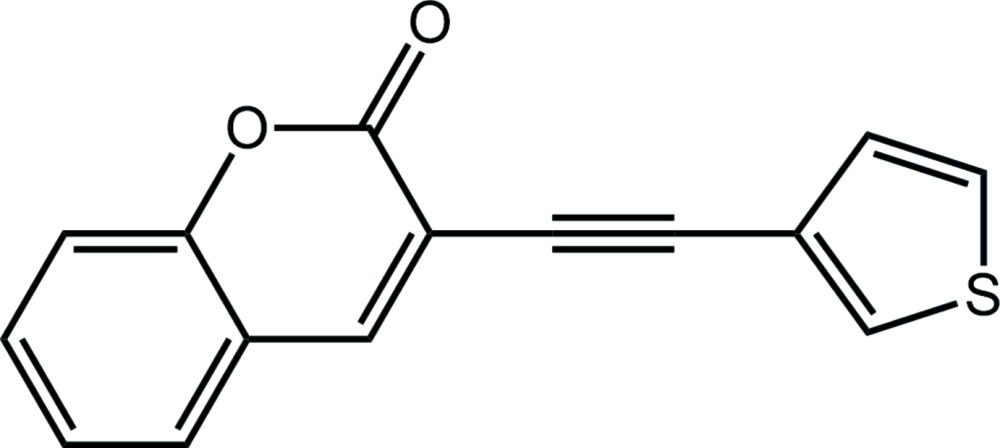


## Experimental   

### Crystal data  

C_15_H_8_O_2_S
*M* *_r_* = 252.27
Monoclinic, 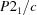

*a* = 10.7726 (6) Å
*b* = 9.7572 (3) Å
*c* = 12.2084 (5) Å
β = 115.547 (6)°
*V* = 1157.77 (11) Å^3^
*Z* = 4
Cu *K*α radiation
μ = 2.40 mm^−1^
*T* = 100 K
0.25 × 0.15 × 0.05 mm

### Data collection  

Agilent CCD diffractometer
Absorption correction: multi-scan (*CrysAlis PRO*; Agilent, 2011 ▸) *T*_min_ = 0.338, *T*_max_ = 1.000
4511 measured reflections
2373 independent reflections
2108 reflections with *I* > 2σ(*I*)
*R*_int_ = 0.023

### Refinement  

*R*[*F* ^2^ > 2σ(*F* ^2^)] = 0.050
*wR*(*F* ^2^) = 0.156
*S* = 1.06
2373 reflections
163 parameters
H-atom parameters constrained
Δρ_max_ = 0.42 e Å^−3^
Δρ_min_ = −0.57 e Å^−3^

### 

Data collection: *CrysAlis PRO* (Agilent, 2011 ▸); cell refinement: *CrysAlis PRO*; data reduction: *CrysAlis PRO*; program(s) used to solve structure: *SIR2014* (Burla *et al.*, 2015 ▸); program(s) used to refine structure: *SHELXL2014* (Sheldrick, 2015 ▸); molecular graphics: *ORTEP-3 for Windows* (Farrugia, 2012 ▸) and *DIAMOND* (Brandenburg, 2006 ▸); software used to prepare material for publication: *MarvinSketch* (ChemAxon, 2010 ▸) and *publCIF* (Westrip, 2010 ▸).

## Supplementary Material

Crystal structure: contains datablock(s) I, New_Global_Publ_Block. DOI: 10.1107/S2056989015002157/su5073sup1.cif

Structure factors: contains datablock(s) I. DOI: 10.1107/S2056989015002157/su5073Isup2.hkl

Supporting information file. DOI: 10.1107/S2056989015002157/su5073Isup3.cml

**Fig. 1.**
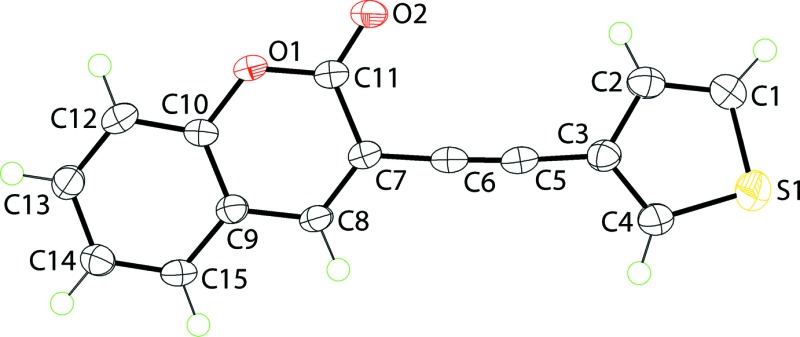
Molecular structure of the title compound showing atom labelling and displacement ellipsoids at the 70% probability level.

**Fig. 2.**
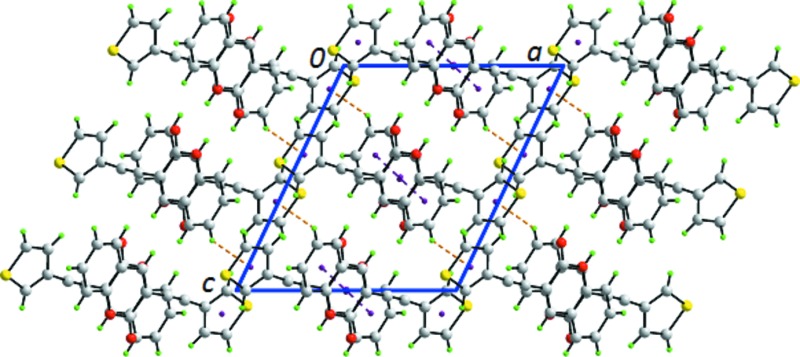
A view in projection down the b axis of the unit-cell contents. The π–π and C—H···π interactions are shown as purple and orange dashed lines, respectively.

. DOI: 10.1107/S2056989015002157/su5073fig1.tif
Mol­ecular structure of the title compound showing atom labelling and displacement ellipsoids at the 70% probability level.

b . DOI: 10.1107/S2056989015002157/su5073fig2.tif
A view in projection down the *b* axis of the unit-cell contents. The π–π and C—H⋯π inter­actions are shown as purple and orange dashed lines, respectively.

CCDC reference: 1046686

Additional supporting information:  crystallographic information; 3D view; checkCIF report

## Figures and Tables

**Table 1:** Hydrogen-bond geometry (, ) *Cg*1 is the centroid of ring S1,C1C4.

*D*H*A*	*D*H	H*A*	*D* *A*	*D*H*A*
C14H14*Cg*1^i^	0.95	2.89	3.701(2)	144

Symmetry code: (i) 
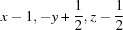
.

## supplementary crystallographic information

### S1. Synthesis and crystallization

The title compound was prepared as per Gueogjian (2011). 3-Bromo coumarin (112.5 mg, 0.5 mmol), potassium tri­fluoro­borate salt (0.55 mmol), PdCl_2_ (dppf).CH_2_Cl_2_ (41 mg, 10 mol%),*i*-Pr_2_NEt (0.3 mL, 1.5 mmol) and 1,4-dioxane/H_2_O (2/1, 3 mL), in aceto­nitrile (20 mL) were added to a two-necked round-bottomed flask equipped with a reflux condenser under N_2_. The reaction mixture was heated under reflux at 353 K, and was monitored by TLC and GC analysis. After the consumption of the 3-bromo­coumarin, the mixture was extracted twice with ethyl acetate (50 mL). The organic phase was separated, dried over MgSO_4_ and concentrated under vacuum. The residue was purified by flash chromatography (ethyl acetate/hexane 10:90). The title compound was obtained as a dark-yellow solid in 53% yield. Suitable crystals were obtained by slow evaporation from a mixture of ethyl acetate/hexane.

### S2. Refinement

C-bound H-atoms were placed in calculated positions (C—H = 0.95 Å) and were included in the refinement in the riding model approximation, with *U_iso_*(H) = 1.2*U_eq_*(C).

### S3. Comment

Coumarins are heterocycles presenting a wide range of different biological activities (Wu *et al.*, 2009; Roussaki *et al.*, 2014). As part of our on-going interest in the synthesis and crystal structures of coumarin derivatives with biological activity (Stefani *et al.*, 2012; Caracelli *et al.*, 2015) the title compound was synthesized (Gueogjian, 2011).

### S4. Experimental

The title compound was prepared as per Gueogjian (2011). 3-Bromo coumarin (112.5 mg, 0.5 mmol), potassium trifluoroborate salt (0.55 mmol), PdCl_2_ (dppf)·CH_2_Cl_2_ (41 mg, 10 mol%),*i*-Pr_2_NEt (0.3 ml, 1.5 mmol) and 1,4-dioxane/H_2_O (2/1, 3 ml), in acetonitrile (20 ml) were added to a two-necked round-bottomed flask equipped with a reflux condenser under N_2_. The reaction mixture was heated under reflux at 353 K, and was monitored by TLC and GC analysis. After the consumption of the 3-bromocoumarin, the mixture was extracted twice with ethyl acetate (50 ml). The organic phase was separated, dried over MgSO_4_ and concentrated under vacuum. The residue was purified by flash chromatography (ethyl acetate/hexane 10:90). The title compound was obtained as a dark-yellow solid in 53% yield. Suitable crystals were obtained by slow evaporation from a mixture of ethyl acetate/hexane.

### Crystal data

**Table:**

C_15_H_8_O_2_S	*F*(000) = 520
*M**_r_* = 252.27	*D*_x_ = 1.447 Mg m^−^^3^
Monoclinic, *P*2_1_/*c*	Cu *K*α radiation, λ = 1.54184 Å
*a* = 10.7726 (6) Å	Cell parameters from 2362 reflections
*b* = 9.7572 (3) Å	θ = 4.0–76.0°
*c* = 12.2084 (5) Å	µ = 2.40 mm^−^^1^
β = 115.547 (6)°	*T* = 100 K
*V* = 1157.77 (11) Å^3^	Prism, dark yellow
*Z* = 4	0.25 × 0.15 × 0.05 mm

### Data collection

**Table:**

Agilent CCD diffractometer	2108 reflections with *I* > 2σ(*I*)
Radiation source: SuperNova (Cu) X-ray Source	*R*_int_ = 0.023
ω scans	θ_max_ = 76.2°, θ_min_ = 4.6°
Absorption correction: multi-scan (*CrysAlis PRO*; Agilent, 2011)	*h* = −13→11
*T*_min_ = 0.338, *T*_max_ = 1.000	*k* = −12→10
4511 measured reflections	*l* = −15→14
2373 independent reflections	

### Refinement

**Table:**

Refinement on *F*^2^	Primary atom site location: structure-invariant direct methods
Least-squares matrix: full	Hydrogen site location: inferred from neighbouring sites
*R*[*F*^2^ > 2σ(*F*^2^)] = 0.050	H-atom parameters constrained
*wR*(*F*^2^) = 0.156	*w* = 1/[σ^2^(*F*_o_^2^) + (0.1031*P*)^2^ + 0.5663*P*] where *P* = (*F*_o_^2^ + 2*F*_c_^2^)/3
*S* = 1.06	(Δ/σ)_max_ < 0.001
2373 reflections	Δρ_max_ = 0.42 e Å^−^^3^
163 parameters	Δρ_min_ = −0.57 e Å^−^^3^
0 restraints	

### Special details

**Table:**

Geometry. All e.s.d.'s (except the e.s.d. in the dihedral angle between two l.s. planes) are estimated using the full covariance matrix. The cell e.s.d.'s are taken into account individually in the estimation of e.s.d.'s in distances, angles and torsion angles; correlations between e.s.d.'s in cell parameters are only used when they are defined by crystal symmetry. An approximate (isotropic) treatment of cell e.s.d.'s is used for estimating e.s.d.'s involving l.s. planes.

### Fractional atomic coordinates and isotropic or equivalent isotropic displacement parameters (Å^2^)

**Table:**

	*x*	*y*	*z*	*U*_iso_*/*U*_eq_	
S1	1.08150 (6)	0.79946 (6)	0.56811 (5)	0.0338 (2)	
O1	0.47823 (14)	0.23152 (14)	0.60599 (12)	0.0194 (3)	
O2	0.62583 (14)	0.39103 (15)	0.71228 (12)	0.0236 (3)	
C1	1.0505 (2)	0.80962 (19)	0.69506 (19)	0.0225 (4)	
H1	1.0976	0.8684	0.7624	0.027*	
C2	0.9466 (2)	0.7191 (2)	0.68413 (19)	0.0236 (4)	
H2	0.9143	0.7081	0.7448	0.028*	
C3	0.8928 (2)	0.64361 (19)	0.57262 (18)	0.0204 (4)	
C4	0.9581 (2)	0.6785 (2)	0.50099 (19)	0.0274 (5)	
H4	0.9368	0.6396	0.4236	0.033*	
C5	0.7859 (2)	0.5442 (2)	0.54250 (17)	0.0207 (4)	
C6	0.69803 (19)	0.4614 (2)	0.52352 (16)	0.0198 (4)	
C11	0.57073 (19)	0.3326 (2)	0.61608 (17)	0.0186 (4)	
C7	0.59579 (19)	0.35987 (19)	0.50841 (17)	0.0183 (4)	
C8	0.5239 (2)	0.29061 (19)	0.40315 (18)	0.0194 (4)	
H8	0.5380	0.3115	0.3334	0.023*	
C9	0.4272 (2)	0.18649 (19)	0.39642 (18)	0.0183 (4)	
C15	0.3539 (2)	0.1072 (2)	0.29190 (17)	0.0211 (4)	
H15	0.3642	0.1249	0.2198	0.025*	
C14	0.2672 (2)	0.00402 (19)	0.29346 (18)	0.0216 (4)	
H14	0.2187	−0.0497	0.2229	0.026*	
C13	0.2510 (2)	−0.0214 (2)	0.39989 (18)	0.0224 (4)	
H13	0.1905	−0.0920	0.4004	0.027*	
C12	0.3219 (2)	0.0550 (2)	0.50393 (18)	0.0218 (4)	
H12	0.3114	0.0371	0.5759	0.026*	
C10	0.40856 (19)	0.1581 (2)	0.50072 (17)	0.0185 (4)	

### Atomic displacement parameters (Å^2^)

**Table:**

	*U*^11^	*U*^22^	*U*^33^	*U*^12^	*U*^13^	*U*^23^
S1	0.0356 (4)	0.0357 (4)	0.0303 (4)	−0.0118 (2)	0.0144 (3)	0.0005 (2)
O1	0.0231 (7)	0.0234 (7)	0.0148 (6)	−0.0009 (5)	0.0109 (5)	0.0002 (5)
O2	0.0262 (7)	0.0299 (8)	0.0159 (7)	−0.0019 (6)	0.0102 (6)	−0.0023 (6)
C1	0.0212 (9)	0.0219 (9)	0.0222 (10)	0.0033 (7)	0.0072 (8)	−0.0011 (7)
C2	0.0254 (10)	0.0257 (9)	0.0209 (10)	0.0022 (8)	0.0112 (8)	−0.0019 (7)
C3	0.0221 (9)	0.0205 (9)	0.0182 (9)	0.0010 (7)	0.0082 (7)	0.0021 (7)
C4	0.0334 (11)	0.0304 (10)	0.0194 (10)	−0.0080 (9)	0.0122 (9)	−0.0008 (8)
C5	0.0244 (9)	0.0241 (9)	0.0154 (8)	0.0034 (8)	0.0104 (7)	0.0018 (7)
C6	0.0246 (10)	0.0218 (9)	0.0142 (8)	0.0040 (7)	0.0096 (7)	0.0005 (7)
C11	0.0211 (9)	0.0202 (9)	0.0159 (9)	0.0030 (7)	0.0092 (7)	0.0009 (7)
C7	0.0208 (9)	0.0197 (9)	0.0164 (9)	0.0016 (7)	0.0099 (7)	0.0016 (7)
C8	0.0228 (9)	0.0226 (9)	0.0160 (9)	0.0000 (7)	0.0113 (8)	0.0006 (7)
C9	0.0207 (9)	0.0183 (8)	0.0174 (9)	0.0011 (7)	0.0098 (7)	0.0002 (7)
C15	0.0245 (9)	0.0255 (9)	0.0146 (8)	0.0006 (7)	0.0097 (7)	−0.0003 (7)
C14	0.0238 (9)	0.0213 (9)	0.0194 (9)	0.0002 (7)	0.0092 (7)	−0.0020 (7)
C13	0.0227 (9)	0.0230 (9)	0.0224 (10)	−0.0020 (7)	0.0105 (8)	0.0014 (7)
C12	0.0255 (10)	0.0235 (9)	0.0198 (9)	0.0026 (8)	0.0130 (8)	0.0048 (7)
C10	0.0210 (9)	0.0200 (9)	0.0148 (9)	0.0019 (7)	0.0081 (7)	−0.0008 (7)

### Geometric parameters (Å, º)

**Table:**

S1—C4	1.701 (2)	C11—C7	1.476 (3)
S1—C1	1.723 (2)	C7—C8	1.360 (3)
O1—C11	1.369 (2)	C8—C9	1.432 (3)
O1—C10	1.377 (2)	C8—H8	0.9500
O2—C11	1.206 (2)	C9—C10	1.400 (3)
C1—C2	1.387 (3)	C9—C15	1.408 (3)
C1—H1	0.9500	C15—C14	1.378 (3)
C2—C3	1.432 (3)	C15—H15	0.9500
C2—H2	0.9500	C14—C13	1.406 (3)
C3—C4	1.381 (3)	C14—H14	0.9500
C3—C5	1.426 (3)	C13—C12	1.384 (3)
C4—H4	0.9500	C13—H13	0.9500
C5—C6	1.189 (3)	C12—C10	1.384 (3)
C6—C7	1.433 (3)	C12—H12	0.9500
			
C4—S1—C1	93.39 (10)	C7—C8—C9	120.69 (18)
C11—O1—C10	122.76 (15)	C7—C8—H8	119.7
C2—C1—S1	109.76 (15)	C9—C8—H8	119.7
C2—C1—H1	125.1	C10—C9—C15	118.13 (18)
S1—C1—H1	125.1	C10—C9—C8	118.32 (18)
C1—C2—C3	113.49 (19)	C15—C9—C8	123.50 (18)
C1—C2—H2	123.3	C14—C15—C9	120.50 (17)
C3—C2—H2	123.3	C14—C15—H15	119.8
C4—C3—C5	125.39 (18)	C9—C15—H15	119.8
C4—C3—C2	111.53 (18)	C15—C14—C13	119.78 (18)
C5—C3—C2	123.08 (18)	C15—C14—H14	120.1
C3—C4—S1	111.83 (16)	C13—C14—H14	120.1
C3—C4—H4	124.1	C12—C13—C14	120.90 (18)
S1—C4—H4	124.1	C12—C13—H13	119.6
C6—C5—C3	176.60 (19)	C14—C13—H13	119.6
C5—C6—C7	176.52 (19)	C13—C12—C10	118.53 (17)
O2—C11—O1	117.48 (17)	C13—C12—H12	120.7
O2—C11—C7	125.43 (18)	C10—C12—H12	120.7
O1—C11—C7	117.09 (16)	O1—C10—C12	117.02 (16)
C8—C7—C6	123.92 (17)	O1—C10—C9	120.80 (17)
C8—C7—C11	120.25 (17)	C12—C10—C9	122.17 (18)
C6—C7—C11	115.83 (16)		
			
C4—S1—C1—C2	0.47 (17)	C7—C8—C9—C10	0.1 (3)
S1—C1—C2—C3	−0.5 (2)	C7—C8—C9—C15	−177.24 (18)
C1—C2—C3—C4	0.3 (3)	C10—C9—C15—C14	−0.5 (3)
C1—C2—C3—C5	179.59 (18)	C8—C9—C15—C14	176.84 (18)
C5—C3—C4—S1	−179.21 (16)	C9—C15—C14—C13	0.6 (3)
C2—C3—C4—S1	0.1 (2)	C15—C14—C13—C12	−0.6 (3)
C1—S1—C4—C3	−0.30 (18)	C14—C13—C12—C10	0.5 (3)
C10—O1—C11—O2	179.74 (16)	C11—O1—C10—C12	177.52 (16)
C10—O1—C11—C7	−0.9 (3)	C11—O1—C10—C9	−1.6 (3)
O2—C11—C7—C8	−177.71 (19)	C13—C12—C10—O1	−179.57 (16)
O1—C11—C7—C8	3.0 (3)	C13—C12—C10—C9	−0.5 (3)
O2—C11—C7—C6	2.3 (3)	C15—C9—C10—O1	179.50 (16)
O1—C11—C7—C6	−177.02 (15)	C8—C9—C10—O1	2.0 (3)
C6—C7—C8—C9	177.45 (17)	C15—C9—C10—C12	0.5 (3)
C11—C7—C8—C9	−2.6 (3)	C8—C9—C10—C12	−177.01 (17)

### Hydrogen-bond geometry (Å, º)

Cg1 is the centroid of ring S1,C1···C4.

**Table:**

*D*—H···*A*	*D*—H	H···*A*	*D*···*A*	*D*—H···*A*
C14—H14···*Cg*1^i^	0.95	2.89	3.701 (2)	144

Symmetry code: (i) *x*−1, −*y*+1/2, *z*−1/2.
