# Supplementary figures and images for: Crystal structure of 3-[2-(thio­phen-3-yl)ethyn­yl]-2H-chromen-2-one
Source: Acta Crystallogr E Crystallogr Commun. 2015 Feb 7;71(Pt 3):o154–5. doi: 10.1107/S2056989015002157 (PMC4350713; doi:10.1107/S2056989015002157)

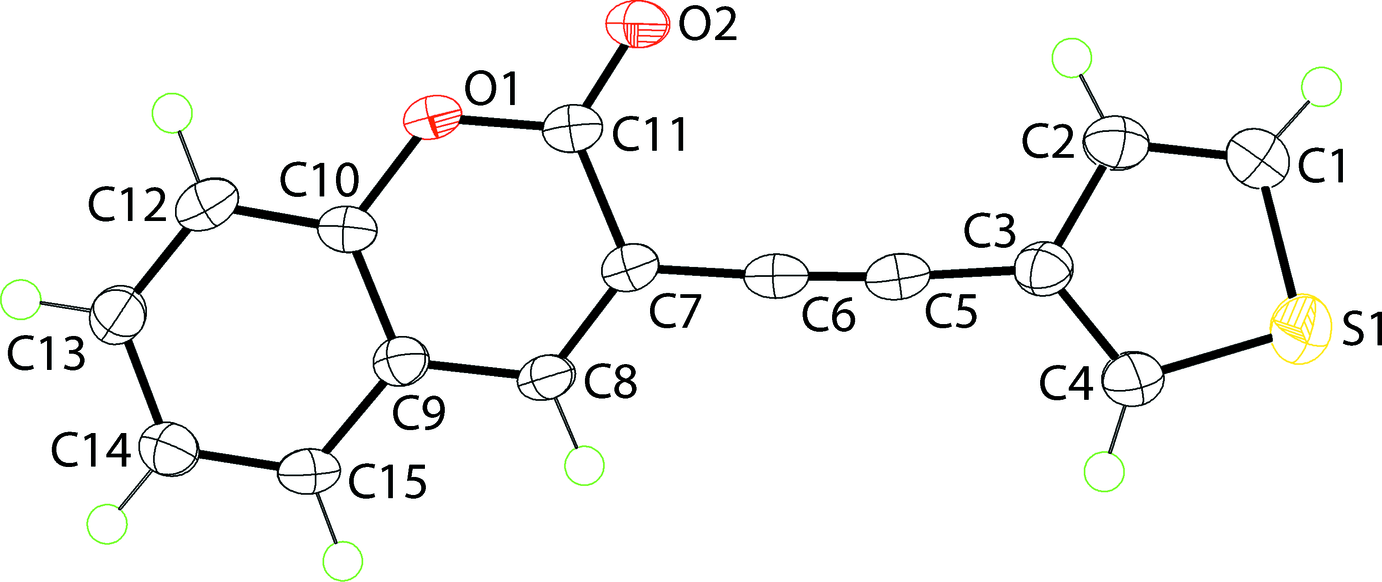

Supplement: Supplementary file 4 [file e-71-0o154-fig1.tif]

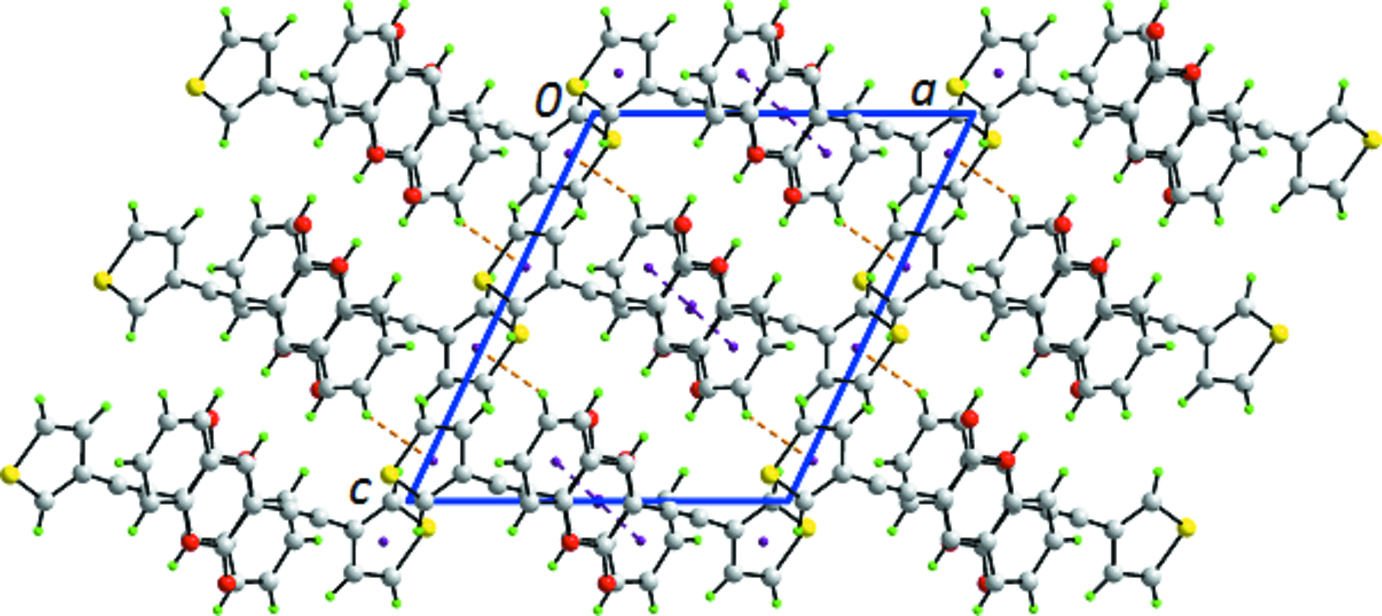

Supplement: Supplementary file 5 [file e-71-0o154-fig2.tif]
